# The interplay between ethics, justice, corporate social responsibility, and performance management sustainability

**DOI:** 10.3389/fpsyg.2024.1323910

**Published:** 2024-02-29

**Authors:** Aharon Tziner, Menachem Persoff

**Affiliations:** ^1^Peres Academic Center, Rehovot, Israel; ^2^Tel-Hai Academic College, Upper Galilee, Israel

**Keywords:** performance management and ethics, performance management and organizational justice, performance management and corporate social responsibility, performance appraisal, contextual factors and performance management

## Abstract

In the increasingly volatile 21st century globalized and interconnected business landscape, organizations face increasing scrutiny concerning their ethical behavior, social responsibilities, and overall performance. This paper looks at some of the factors that link the notions of ethics, justice, and Corporate Social Responsibility (CSR), with an eye to their theoretical underpinnings and complexities and their relationship to the efficient and sustainable operation of Sustainable Performance Management (with special emphasis on CSR). Drawing on theoretical foundations and empirical evidence, we provide practical recommendations for organizations to promote ethics, justice, CSR, and effective and sustainable performance management. Suggestions include fostering ethical leadership by modeling ethical behavior and promoting ethical decision-making. We believe that the suggested practical measures may bridge the gap between academic perspectives and the practical realities of ensuring favorable, sustainable, work climates and work processes.

## Introduction

Ever since the emergence of humanity on this planet, work has been extremely challenging. So, how best should a person toil, and how would one measure that individual’s success? While a quantitative response appears logical—the degree to which outcomes serve the artisan and, where appropriate, his or her clients—the answer to this question is by no means clear. Much, for example, depends on the economic climate and the question of supply and demand. The output is also a function of the efficacy of the manufacturing process or the efficiency of service providers. Or perhaps the criterion for individual performance differs from team performance (Martell and Guzzo, 1991; cited in Fein, 2022). Moreover, as work conditions and market trends evolve (e.g., non-traditional work arrangements, digital communication, increased work task complexity, global team performance), the criteria of successful performance change concomitantly (Fein, 2022). Finally, one might argue that sufficient success criteria in one culture defy consideration in another (e.g., Natukunda, 2022).

Performance and performance appraisal are often subjective. As some research on the subject indicates, success is primarily a product of the eye of the beholder. For instance, we might ask, were the *a priori* work objectives clarified? Were the working conditions appropriate for the tasks to be fulfilled? Were the appraisers themselves sufficiently motivated and skilled to do *their* job? Were the assessors biased in their ratings? Were the measuring tools reliable and consistent over time? See Tziner and Rabenu (2018) for a fuller account of the issues of performance appraisal.

Productivity (among other factors) is related to the work environment that incorporates the relationships developed between employees and their supervisors (Roch, 2015)—and, in today’s evolving work climate, increasingly to the preferences and demands of consumers and the rising Z generation (Rabenu, 2021)—the upholding of ethical and judicial standards in the organization is increasingly recognized as an essential feature that should characterize both firms and the larger corporations whose concerns reach beyond the bottom-line to corporate social responsibility, now a catchword in 21st-century economics (Vevere and Svirina, 2020).

In the increasingly turbulent 21st-century globalized and interconnected business landscape, organizations face increasing scrutiny regarding their ethical behavior, social responsibilities, and overall performance. Ethics and justice are essential aspects of creating a fair and morally responsible organizational culture, while corporate social responsibility encompasses an organization’s commitment to societal welfare. Within that context, performance management serves as a crucial framework for assessing and optimizing organizational effectiveness. Understanding the interplay between ethics, justice, CSR, and performance management is vital for organizations to establish a strong ethical foundation, foster a just work environment, fulfill societal obligations, and drive performance.

With this backdrop, we propose to look (somewhat parsimoniously) at notions of ethics, justice, and CSR with an eye to their theoretical underpinnings, complexities, and relationship to the efficient operation of performance management at the individual, group, and firm levels with special emphasis on CSR.

The objective of this article is to dwell theoretically upon the intricacies and the interplay between ethical conduct in business, fair treatment in the workplace, socially responsible practices (CSR), and sustained performance management. The authors did not intend to report results based on an empirical study, but to review the extant literature. The suggested practical measures take some steps to bridge the gap between academic perspectives and the practical realities of organizations. By implementing these practical measures, organizations can enhance their sustainable performance management processes, promote ethical behavior, fairness, and social responsibility, and create a positive work environment that fosters employee engagement, growth, and organizational success. The novelty consists of attempting to shed light on how the concepts might link and foster vital sustained performance management.

We begin our odyssey by briefly reviewing the elements under discussion: Performance Management (PM), Ethics, Justice, and Corporate Social Responsibility (CSR).

## Performance management

Performance management encompasses the processes and systems used to measure, monitor, and enhance individual, group, departmental, firm, and corporate organizational performance. In essence, PM involves setting performance expectations and planning work, developing the capacity to perform, continual performance monitoring and provision of feedback, identifying development needs, and (re) aligning individual and team goals with organizational objectives, serving as a framework for improving productivity, fostering employee engagement, and driving overall organizational effectiveness.

Rather than emphasizing the bottom line, Tziner and Rabenu (2018, p. 5) interestingly highlighted the holistic and human element when they defined PM as “the creation of an entire system (a setting, work environment, culture), bringing together all the essential factors so that all of the people involved in the organization can work in an aligned and coordinated fashion to the best of their abilities” (after Grubb, 2007, p. 2). In essence, these researchers paid more attention to the process and dynamics of PM than the outcomes of performance management. Understandably, because work performance requirements constantly change due to changes in a gamut of factors such as the nature of work, work legislation, economic conditions, customer demands, and so forth, the criteria for effective and efficient performance may change over time (Dalal et al., 2020). Thus, in addition to any retrospective appraisals, performance management should better look to improvements on the job and comprise actions such as setting specific work goals and performance standards, providing feedback about performance, and determining training and development targets for employees (Briscoe and Claus, 2008; Aguinis, 2013; DeNisi and Smith, 2014; Natukunda, 2022).

### Theoretical underpinnings

PM is informed by several theories that include:

#### Goal-setting theory

Attributed to Edwin Locke, goal setting theory, as its name implies, stresses the significance of clear goal setting. The goals should be precise and attainable; as such, they will enhance an individual’s motivation, which, in turn, enhances performance. Moreover, feedback and continuous monitoring enhance progress toward the set goals.

#### Expectancy theory

Expectancy theory posits that individuals’ efforts and performance are influenced by their expectations of outcomes. According to the theory, employees will try harder when they deem that doing so will lead to the required performance and that, moreover, the outcomes will be rewarded. In short, PM management practices aligned with expectancy theory principles ensure that employees understand the link between their efforts, performance expectations, and rewards.

#### Social cognitive theory

The theory, which found its roots in social learning theory (e.g., Bandura, 1977), highlights how individuals observe and learn from other people’s behaviors and experiences, and how their belief in their ability to perform tasks successfully (self-efficacy) motivates them to engage in beneficial performance behaviors. Thus, PM leverages social cognitive theory principles by providing role modeling, coaching, and skill development opportunities to enhance self-efficacy and performance.

### Aspects of PM

Traditionally, performance appraisal (PA) and (annual) feedback constituted the integral components of performance management. Appraisal systems provide a formal mechanism for evaluating and assessing an individual’s performance against set goals and expectations. Indeed, Colquitt et al. (2001) highlighted the importance of fair and accurate feedback in enhancing employee performance and organizational effectiveness. However, in the not-too-distant past, much dissatisfaction was expressed with PA. For example, contended that managers and subordinates disdain performance ratings because they feel these do not accurately reflect actual performance because of many issues, including the reliability of its measuring instruments, appraiser bias, and its stressful effects on employees and appraisers alike. Indeed, ratees experience frustration because the ratings are not proportional to the improvement of performance; they are narrow, focused on retrospective performance, critical, and judgmental.

It is important to stress that despite their drawbacks, the PM world has not given up on PA systems. Indeed, an effective PM system should focus on evaluating past performance *and* fostering continuous improvement and development. Notably, while several corporations, such as Dell, Deloitte, and Microsoft, abandoned PA, other concerns reintroduced the system for no reason other than their employees wanted some indication of their performance. Furthermore, Tziner and Rabenu (2018) observed that the traditional PA paradigms have added value insofar as they provide important data for administration purposes and record keeping.

While Tziner and Roch (2016) would endorse the systems’ inherent impairments, they, nevertheless, recommended several practical approaches to fixing the system, including (as indicated) focusing on the process rather than on results, in line with Murphy and Cleveland’s (1995) reservations concerning PA. Tziner and Roch (2016) further indicated that following Roch et al. (2007), relative formats that compare individuals to peers are considered less fair than absolute formats comparing individuals to standards. This observation has consequences; it positively influences perceived interpersonal justice, especially if employees do not trust their supervisors (Roch, 2015). In turn, there are notable implications for job satisfaction, organizational commitment, task performance, and organizational citizenship behaviors.

Moreover, Rabenu and Tziner (2015) advocated creating a personalized performance appraisal based on employees’ knowledge, skills, abilities, and other personal qualities (KSAO). Notwithstanding the value of traditional PA for administrative purposes, Rabenu and Tziner (2016) posited that performance appraisals should be customized to fit the specific job *and* the employee’s characteristics insofar as they impact the individual’s work performance. Such customized appraisals, already in vogue (Kluger and Nir, 2010; Bouskila-Yam and Kluger, 2011), stress employees’ successes at work (strength-based performance) and how to build upon them in the future. This ‘optimistic’ approach to work appraisal would most surely enhance employees’ motivation and performance and minimize negative perceptions of procedural organizational justice and legal compliance (see below).

Tziner and Roch (2016) further argued that effective rater training also matters, especially frame of reference training (FOR), shown to enhance rating accuracy (Woehr and Huffcutt, 1994; Roch et al., 2012) by reducing rating distortions (due to personal agendas) and providing both raters and ratees with a common definition of performance. Notably, in a survey of 101 US firms, FOR training was the most applied among the 76% of 101 firms studied that used rater training (Gorman et al., 2015). Tziner and Roch (2016) concluded that, if nothing else, rater training can improve perceived performance appraisal effectiveness, which is more likely to enhance employee performance.

Furthermore, notwithstanding reservations that authentic feedback might generate negative reactions (e.g., Bouskila-Yam and Kluger, 2011) and problems for managers (Brown et al., 2016; see also Maley et al., 2021), Tziner et al. (2020) advocated for punctual and credible feedback, a recommendation that frequently appears in the literature (e.g., McCarthy and Garavan, 2007; Aguinis et al., 2012; Maley and Kramer, 2014; Pulakos et al., 2019). Rather than wait with increasing anxiety for the end-of-the-year assessment, employees welcome a good word and encouragement or suggestions for improvement in one-to-one informal settings at various times during the year.

Furthermore, Fein (2022) has cautioned that performance management activities, such as the extensive collection of multi-source feedback, very frequent formal revisions of goals, and frequent periodic performance management meetings, do not always achieve positive results considering the time and effort expended.

In that context, DeNisi and Smith (2014) recommended that, *inter alia,* ongoing PM reforms should streamline aspects of job performance that can be measured using automated data to achieve more rapid and timely evaluations (cf. Pulakos et al., 2019), a recommendation increasingly adopted with the employment of performance management software and digital platforms to streamline PM processes. These tools include features such as goal tracking, continuous feedback, performance analytics, and 360-degree feedback, enabling more efficient and data-driven performance management practices.

### Current trends in PM

Given space limitations, we briefly sum up further trends in the PM world.

Maley et al. (2021) indicated that PM literature was largely geared toward firm-level analysis over 20 years (from 1998 to 2018). Attention was primarily focused on the PM’s objectives so that they be clear to all stakeholders and reflect the firm’s strategic goals (e.g., Maley and Kramer, 2014; Iqbal et al., 2015). In that respect, PM increasingly emphasizes goal alignment and the use of Objectives and Key Results (OKRs) frameworks, whereby OKRs provide a clear and transparent way to set and track goals, ensuring that individual and team objectives align with organizational priorities and contribute to overall success.

However, with the greater recent recognition of employees’ unique needs and preferences, PM personnel are moving toward individualized performance management approaches, including customized performance goals, feedback styles, skill-building programs, and development plans to suit individual strengths, motivations, and career aspirations.

In addition, the use of data analytics and predictive analytics is becoming more prevalent in performance management. Organizations are leveraging data to analyze performance trends, identify areas for improvement, and make data-informed decisions related to talent development, succession planning, and performance interventions.

Furthermore, performance management systems increasingly include performance-based incentives and rewards to recognize and reinforce high performance. These may include financial bonuses, promotions, recognition programs, and non-monetary incentives. Performance-based rewards provide motivation, reinforce desired behaviors, and signal the value placed on performance excellence. Additionally, there is a growing recognition of the connection between employee well-being and performance management. Organizations are incorporating well-being initiatives, such as stress management programs, work-life balance support, and mental health resources, into their performance management processes to foster employee resilience and optimize performance.

Finally, Diversity, Equity, and Inclusion (DEI) considerations are gaining prominence in performance management practices. Organizations strive to ensure fairness, mitigate bias, and promote inclusivity in performance evaluation, goal setting, and feedback processes, aiming to create equitable opportunities for all employees.

Viewing the future of PM, DeNisi and Smith (2014) described a “bundle of HR practices” that promote skills, motivation, and opportunities that form part of the entire repertoire of performance management—of which PA is but one aspect when properly applied. Informed, appropriate, and careful employment of these best practices—“salient, visible, valid, legitimate, relevant, consistent, instrumental, and fair”—would likely empower and motivate employees to leverage KSAO for the organization’s benefit.

The goal is to create a “climate of performance” in which informal approaches complement traditional formal systems, including personalized mentoring, off-the-cuff encounters with supervisors, staff social events and commemorations, and open communication channels with employees (Armstrong, 2017).

Moreover, DeNisi and Smith (2014, p. 147) advocate that management select those practices that merge with their organizational goals and policies and then synergize them with the entire HR system that incorporates training, reward systems, job design systems, and so on, impacting performance at the individual, team, and firm levels.

Notably, the informal aspects of PM appear to be popular and decisive. Among its many advantages, informality in the workplace (such as informal, spontaneous, face-to-face personal feedback or an open, colleague-friendly political climate) breaks down employee cynicism, mistrust in management, and perceived injustice of performance ratings while enhancing LMX and OCB, among other outcomes that increase motivation, loyalty, and cooperation with the upper realms of management and identification with their organizational policies and strategy. The latter outcomes have further been indicated to enhance the firm’s productivity with all the benefits to the organization, such as net profits, less turnover, sustainability, and public image.

### Challenges

Challenges, however, face organizations trying to assess performance in today’s world where work styles and work values are rapidly changing as reflected in the gig community, the Y and Z generational collaborative work styles, OI and other transformative technologies, diversity in the workforce, and the increasing tendency to work at home—all phenomena that are shaping the values, preferences, and demands of workers in today’s working climate. Management and HR personnel, burdened with a plethora of tasks and faced with numerous recommendations emerging from the literature, need to focus and create a working strategy of performance management that matches their firms’ specific goals and policies while maintaining flexibility and openness to review PM strategies as the job market evolves (see below).

Having described these elements, one needs to reengage Fein’s (2022) insights into the discussion. To expand on his observation above that the cost of managerial time and effort expended by employees in engaging in complex performance management activities can serve as a disincentive, Fein indicates further that they can be perceived as unfair (Bauwens et al., 2019), induce cynicism among staff members, and lead to burnout (Gabris and Ihrke, 2001).

A further dilemma facing both academia and practitioners is how PM is measured. What are the appropriate criteria, and what constitutes success? Is success measured by employee turnover, net financial profits, productivity, or contribution to society in the form of sustainability and public health—or any combination of these and other dimensions of success? Furthermore, does “success” incorporate behaviors that go beyond the call of duty, such as OCBs?

Moreover, as indicated, we should ask what the mechanisms and dynamics that engender “success” are. What external and internal factors influence employee behavior and management decision-making, and what factors mediate employee behavior? How can management be better trained and equipped to handle these questions and focus on the various dimensions of PM, from staff selection, KSAO development, and staff training to job design systems, performance appraisal and reward mechanisms, and the promotion of positive work culture at the individual, team, and firm levels?

To that end, Posthuma et al. (2018) invoked evidence-based management (EBMgt) to identify PM behaviors, processes, practical issues, and actions that close the gap between theory and practice, effectively motivating enhanced performance and precluding negative user reactions.

Citing Rousseau (2012), Posthuma et al. (2018) posited that EBMgt capitalizes on “the best scientific evidence, business metrics and indicators, reflective judgment with decision aids, and ethical standards” that consider stakeholder interests (p. 170). Notably, Posthuma and colleagues’ taxonomy integrates mediator and moderator variables into their model, thus ensuring that it “incorporates all the known and important factors that explain the when, why, and how the practices (in the workplace) lead to effective behaviors and positive outcomes” (Posthuma et al., 2018, p. 171).

Furthermore, 50 practices identified by Posthuma et al. (2018) resolved into seven overarching descriptive categories, namely “Strategic Connections, Sound Content, Meticulous Ratings, Professional Administration, Prospective Development, Rich Communication, and Review and Documentation.” The authors suggest that the overall result of the taxonomy provides a “new and unified integration” of diverse literature into a single theoretical framework.

As with DeNisi and Smith’s (2014) formulation of best practices, Posthuma et al. recognized that “not all PM practices are appropriate, affordable, applicable, or necessary” (after Buckingham and Goodall, 2015), but the taxonomy nevertheless offers a variety of practice possibilities. Finally, the taxonomy allows management to adopt PM measures that align well with the organization’s goals, policies and (as indicated) strategies that likely reduce negative employee reactions in the light of that overarching challenge.

Despite the various obstacles, in the theory and implementation of effective performance management practices, organizations can enhance employee performance, foster a culture of accountability and continuous improvement, align individual and team efforts with organizational objectives, and drive overall organizational success. The integration of ethical and justice considerations (see below) into PM further ensures that performance goals and expectations align with ethical principles, promoting a culture of integrity and responsible performance. To these areas, we now turn.

## Ethics

### Ethical theories

Ethical theories provide frameworks for understanding and evaluating ethical decision-making. Within the context of the workplace, business ethics is the study of the moral principles that guide business decision-making. Following the abundant literature and Vevere and Svirina (2020, pp. 64–67), two principal theories of business ethics appear cogent in today’s business world. In brief:

### Deontological theories

Deontological theories focus on the rightness or wrongness of actions, regardless of their consequences. Thus, people should not deviate from the rules; they should follow them. Two major schools of thought emerge from this principle:

#### Kantianism

The theory emphasizes the importance of adhering to universal moral principles. Actions are evaluated based on whether they conform to moral rules or duties. Consequently, based on this notion, everyone should act in the same way.

#### Virtue ethics

In contrast to Kantianism, virtue ethics stresses the development of one’s moral character and cultivating individual and organizational virtues such as honesty, integrity, and courage. Individuals are thus encouraged to strive for excellence in their moral character and make decisions accordingly.

In that spirit, Vevere and Svirina (2020) posited that deontological theories provide a clear and consistent framework for making ethical decisions, help businesses avoid making decisions harmful to others, and build trust and credibility with their stakeholders. On the other hand, the authors asserted that deontological theories can be inflexible and resistant to change, difficult to apply in complex situations, and lead to decisions that are not necessarily in the business’s best interests.

### Consequentialist theories

As implied by its title, the ethical value of any action is judged by its consequences. Some of the most common consequentialist theories in business ethics include:

#### Utilitarianism

This theory postulates that an ethical action is one that engenders the greatest good for the greatest number of people, implying a pay-off of potential benefits of all possible actions against their harmful outcomes on all the people involved. To cite Becker (2019), the ethically right actions are those that produce the greatest net utility.

#### Justice theory

Justice theory holds that the right action is the one that distributes benefits and burdens fairly, which is of relevance in organizations where employees are extremely sensitive to issues of equity.

Vevere and Svirina (2020, p. 65) indicated that while consequentialist theories can help firms make decisions in the “best interests of the greatest number of people”—thus encouraging employees to be productive, in complex situations—the outcomes could be harmful to the minority group and serve a “justified” source of unethical behavior.

Ultimately, the authors contend, the best approach to business ethics is to use a combination of the different theories and to tailor the approach to the specific situation.

## Business ethics

Following the above definitions, business ethics (BE) as a discipline focuses on the principles stakeholders in any business concern should follow in the workplace at the individual, organizational, and societal levels. Vevere and Svirina (2020, p. 65) noted that the field “probes the most appropriate or just designs for firms, markets, market regulations, and political oversight in a democratic society and a globalized economy (Norman, 2013) with emphasis on such issues as normativity, individual-social relations, organizational behavior, and market and political conditions.”

As such, a corporate business must concur with the norms and legal requirements of the host country (*compliance*), examine its quality standards and possibilities of contributing to all the stakeholders and society (*contribution*), and as stressed above, the upshots of their actions (*consequences*). In that respect, two aspects of ethics clash: We might ask in a competitive world—noting how irregular circumstances can ruin an organization—if “business ethics” serves the commercial interest of the firm first, primarily providing the wherewithal to implement ever more successful business strategies, operations, and organizational designs (see Becker, 2019). Or, contrary to this instrumental approach, we ask which utilitarian values are being implemented by the firm for the good of the stakeholders and society, as indicated in the philosophical approach to business ethics.

Ultimately, we concur with Vevere and Svirina (2020) that there are no univocal criteria to determine if an organization is to be regarded as ethical or unethical.

### Components of ethical culture and ethical climate

We now turn to the consideration of the ethical climate in the workplace, which is an outcome of the theories outlined above. Following Victor and Cullen (1988a), when a positive ethical climate is extant, colleagues at work tend to feel good about themselves; they are more likely to bathe in autonomy, trust the organization, and appreciate the organization’s support.

Relating to the theoretical aspects of business ethics, Victor and Cullen (1988a,b) indicate that the ethical climate reflects the shared perceptions about what is morally salient in the business concern, a reflection of the larger organizational culture of norms and practices that pertain in the workplace. Notably, these perceptions relate to *formal elements* of the ethical culture, such as mission statements and a code of ethics, and *informal elements*, including informal communication, language, rituals, and traditions.

In the authors’ parlance, these perceptions give rise to five categories of ethical climates, which reflect, respectively, the motifs underlying the ethical theories described above under the following five categories: (i) *Caring climate*, (ii) *Social-legalistic climate*, (iii) *Organizational-legalistic climate*, (iv) *Instrumental climate*, and (v) *A climate of independence* (Victor and Cullen, 1988a,b).

### Examples of ethical behavior in the workplace

As indicated, in the workplace, ethical considerations and behaviors are diverse and manyfold. For example, management and employees demonstrate honesty and integrity by (a) being truthful and transparent in their interactions and communications, (b) refraining from engaging in deceptive practices, lying, or misrepresenting information, and (c) taking responsibility for one’s actions, admitting mistakes, and maintaining confidentiality when required.

Ethical managers treat colleagues, clients, and stakeholders with respect, fairness, dignity, courtesy, and equality. Employees, likewise, respect diverse perspectives, cultures, and backgrounds and refrain from discriminatory practices. Fairness includes giving everyone an equal opportunity to contribute and recognizing the value of diverse contributions (see Justice below).

Other ethical considerations in the workplace include maintaining confidentiality and privacy, managing conflict of interest, honoring reportage of misconduct and policy violations, and employing efficient use of resources (e.g., time, assets, intellectual property, and promotion of CSR). Perhaps of greatest significance is that management and employees alike follow ethical decision-making processes when faced with complex situations, such as considering the potential impact of decisions on stakeholders, weighing ethical principles and values, and seeking guidance or input when necessary. Ethical decision-making involves being mindful of long-term consequences and striving to make choices that align with ethical standards.

### The effects of ethical behavior on employees’ productivity

The literature is replete with empirical evidence that cultivating a culture that encourages and rewards ethical conduct contributes to a positive work environment, strengthens organizational reputation, and fosters trust among employees and stakeholders. Classical examples include: Treviño et al. (2011), who found that organizations with a strong ethical culture tend to have higher employee commitment, job satisfaction, and performance; Khaltar and Moon (2020), who observed that informal ethics management and transformational leadership reduced unethical behavior and improved organizational commitment in public agencies; Sen and Bhattacharya (2001), who found that companies perceived as ethical and more socially responsible enjoyed better relationships with stakeholders, leading to improved customer loyalty, enhanced brand image, and positive financial performance; Brown et al. (2005) and Mayer et al. (2009), who demonstrated that ethical leaders serve as role models, shaping employee attitudes and behaviors toward ethical conduct.

Ethical leadership positively influences employee trust, commitment, and willingness to engage in ethical behaviors.

## Four issues of concern

### Negative reciprocity

Of particular interest concerning the many laudable studies invoking positive relationships between ethical leadership and performance management is a more recent conceptual paper by Neher and Maley (2019) indicating that those responsible for performance management are themselves not necessarily committed to the task. Indeed, the authors cite evidence of ineffective Ethical Performance Management (EPM) (Pichler et al., 2016; Weibel et al., 2016) attributable to supervisors’ negative feelings toward the process (Elicker et al., 2006) that, in turn, may account for poor EPM wherein the damage outweighs the benefits (Leigh and Watkins, 2010).

In that respect, the authors posit that supervisors’ core value systems may play a part in their levels of commitment, as demonstrated by Bamberger (1986) and Westwood and Posner (1997). Indeed, the authors go so far as to assert that the supervisor’s values fundamentally influence all aspects of employee performance management, contribute to employee performance, and ultimately affect business effectiveness and sustainability.

Consequent to their findings, Neher and Maley (2019, abstract) proposed a “managerial grounded values framework” to serve as a charter “that guides the supervisor’s actions, goals, choices, decisions, and attitudes, principles process and contributes to the effectiveness of the evaluation process and a positive EPM experience that motivates, enhances engagement, and guides personal development.” In short, the more the organization’s espoused values are reflected in the daily running of the firm, the greater the identification of the stakeholders with those values that, in essence, inform the organization’s business ethics with consequent employee productivity and related positive outcomes.

Not surprisingly, the intrinsic values and characteristics of the EPM framework reflect the ethical dimensions and actions that characterize the ethical behaviors indicated above, which have been empirically shown to enhance employee identification with the organization, enhance motivation, and altogether improve employee performance. For example, when employees are aware of the efforts of management to maintain clarity of purpose and fairness in dealing with their issues, they, in turn, enhance their identification with those value-laden ethical behaviors of social exchange theory.

### Emphasis on the bottom line

Another disturbing aspect of business ethics is the recurring emphasis on the bottom line, the instrumental approach to ethics, so often cited as a beneficial outcome of ethical management or performance management. For example, after presenting their circular framework of an effective EPM based on values commitment, Neher and Maley (2019) noted that these core values (e.g., about how to treat employees) and their organizational enactment *are likely to foster sustained superior financial performance* (Barney, 1986; Neher et al., 2018; our emphasis). Or to cite Investopedia (Twin, 2023): “Since that time [the 1960s], the concept of business ethics has evolved. Business ethics goes beyond just a moral code of right and wrong; *it attempts to reconcile what companies must do legally* vs. *maintaining a competitive advantage over other businesses*” (our emphasis). While this may be the case, we ask: Do we treat our employees “nicely” so that we make “the extra buck” or because we value them as human beings, and we value their time and effort as they fulfill their contractual obligations?

In that vein, what are we saying when we talk of the common good or the greatest good to the greatest number of people? Who decides what is good? Unfortunately, we are only too aware of the misuse of this term and how often it has been abused. On the other hand, let us at least discern the basic principles of business ethics that appear to be universally accepted, for example, the 12 espoused by Investopedia (Twin, 2023), namely, leadership, accountability, integrity, respect for others, honesty, respect for laws, responsibility, transparency, compassion, fairness, loyalty, and environmental concern.

Indeed, we would strongly assert that the first step toward improving the ethical aspect of PM should be adopting a code of ethics that sets out the company’s values, ethics, objectives, responsibilities, and business style (e.g., Hoppen, 2002). Notably, involving employees in the process is critical to gaining their support to implement the code.

### Validity of scales

Related to the ethical dimension and stakeholder perceptions of ethical (and fair) procedures is the issue of the validity of the scales used in empirical studies to measure both supervisors’ ethical values and employees’ perceptions of their superiors’ ethical standards and behavior. First, we must consider that each respondent has his or her set of ethical beliefs and standards of morality. Second, we ask to what extent respondents are accurate or true in their responses when, perhaps, they discern among their raters hidden agendas at work. Third, in a changing world where technologies and the marketplace are increasingly at the mercy of political and financial interests, who can say that management’s motives to improve performance are *a priori* pure and utilitarian?

### What about worker ethics?

Last, we might ask why so much attention is directed to management and less to the work values and ethics with which employees enter their work contracts. Indeed, several studies found that workers prefer organizations that promote business ethics (e.g., Treviño et al., 2001). Furthermore, positive relationships were found between job satisfaction and (i) organizational ethics (e.g., Deshpande, 1996) and (ii) high-level workers’ perceived justice (Cohen-Charash and Spector, 2001), the latter positively affecting employees’ organizational commitment (Chen et al., 2010; see below, Justice).

Related somewhat to workers’ preferences, Rabenu (2021), among others, has spelled out that in the twenty-first century, we are gradually entering a workers’ market where the Y and Z generations and the talents are increasingly calling the shots and dictating to employers how to satisfy their needs, such that even universities are catering increasingly toward tailor-made studies. These moves are not intrinsically “bad”—on the contrary—but they illustrate superficially that the old order is dying and that in our competitive and volatile marketplace, “ethics” are increasingly the means and not the end.

## Justice theories

We have referred to perceptions of justice in the workplace partly because justice is related to ethical practices. Indeed, in the context of the workplace, justice theories promote the establishment of fairness and equity in organizations. In brief, the theories that abound are described as follows:

### Distributive justice

Distributive justice pertains to the fair distribution of resources, rewards, and burdens within an organization. Individuals receive what they perceive as their fair share based on relevant criteria such as contribution, need, or merit, through which inequalities are minimized, and equity is achieved in the allocation of resources (e.g., salaries and bonuses) and opportunities (e.g., promotions, benefits, and training).

### Procedural justice

Procedural justice relates to the fairness of decision-making processes within an organization. It emphasizes the importance of transparent, unbiased, and participatory procedures in reaching decisions. Procedural justice suggests that individuals are more likely to perceive decisions as fair when they have a voice in the process, the process is consistent and unbiased, and relevant information is shared openly. Fair procedures contribute to employee trust, satisfaction, and commitment, even if the outcomes of decisions may not always be favorable.

### Interactional justice

Interactional justice focuses on the respectful and dignified treatment of individuals during interactions. *Interpersonal justice* emphasizes treating individuals with dignity, respect, and politeness. It involves giving individuals a voice, listening to their concerns, and showing empathy and sensitivity in interactions. Furthermore, *informational justice* emphasizes providing individuals with clear and timely information that is relevant to the decisions being made. It involves explaining the rationale behind decisions, providing feedback, and ensuring that individuals have access to accurate information that helps them understand the decision-making process.

Justice issues that can arise in the workplace include pay equity, promotion opportunities, performance evaluation, and feedback, distribution of workload and resources, decision-making processes, grievance handling and conflict resolution, diversity and inclusion, and health and safety. By incorporating the principles of justice, organizations can cultivate a work environment that is perceived as fair, equitable, and supportive. In support of this notion, the literature is replete with examples, of which we present a sampling:

A meta-analysis by Smither et al. (1995) found that fairness perception in performance evaluations is positively associated with employee job satisfaction and commitment. Konovsky and Cropanzano (1991) found that perceived unfairness in workload distribution can lead to negative job attitudes and reduced organizational citizenship behaviors, while Breaugh and Starke (2000) indicate that equitable distribution of resources and rewards positively influences employee satisfaction and commitment. Fein et al. (2013) found a positive relationship between perceived interactional justice and levels of leadership-member exchange (LMX) and a positive relationship between ethical climate and LMX. Similarly, Tziner et al. (2015) observed that ethical climate factors, three types of organizational justice dimensions, and LMX were highly interrelated. Zhang et al.’s (2019) investigation of the role of interactional justice and ethical leadership on organizational citizen behavior (OCBO) revealed that interactional justice serves as a conduit that induces employees’ OCBO in response to leaders’ ethical behaviors. Faeq and Ismael (2022), investigating the relationship between organizational justice and job performance using a descriptive-analytic approach, found a positive relationship between organizational justice and job performance, with procedural justice demonstrating the strongest relationship with job performance. Ryan and Saha (2016) suggested that promoting diversity and inclusion in promotion processes can enhance organizational justice perceptions and employee satisfaction.

## Corporate social responsibility

With this background, we now refer to the fourth component of our title, Corporate Social Responsibility (CSR), which refers to an organization’s commitment to conducting business responsibly, both ethically and socially. The organizational actions and initiatives tend to be voluntary and address societal, environmental, and ethical concerns beyond the legal requirements. The organizations employing CSR—primarily large corporations—commit to conducting business in a manner that impacts positively on society and demonstrates accountability toward various stakeholders—employees, customers, communities, and the environment.

CSR is informed by several theories, including *stakeholder theory*, which serves as a foundational concept for understanding CSR. The theory extends beyond the narrow interests of employees and their supervisors, discussed above, and posits that organizations have moral and ethical obligations to consider in the best interests of all the stakeholders affected by the organization, including the customers, suppliers, shareholders, local communities, government bodies, and environmental organizations. Thus, CSR involves managing those relationships and balancing their interests to create long-term value and sustainable outcomes.

CSR initiatives are often further informed by the *Triple Bottom Line*, a comprehensive framework that emphasizes three dimensions of organizational performance: economic, social, and environmental. Beyond financial performance and profits, organizations consider their social and environmental impacts.

Furthermore, based on *Legitimacy Theory*, organizations engage in CSR initiatives to gain and maintain legitimacy in the eyes of stakeholders and society. By aligning their actions with societal expectations and norms, organizations enhance their reputation, build trust, and secure their long-term survival.

Finally, in that vein, *Instrumental Theory* (as noted) posits that CSR initiatives yield tangible benefits for organizations. By addressing societal and environmental concerns, concerns improve competitiveness, attract customers and investors, enhance employee morale and productivity, mitigate risks, and foster long-term profitability.

### CSR practices

CSR practices can bring various benefits to organizations, including enhanced reputation, increased customer loyalty, improved employee engagement, reduced risks, access to new markets, and strengthened stakeholder relationships. Effective CSR initiatives align with the organization’s values, core competencies, and strategic objectives, creating shared value for the organization and society. By addressing societal and environmental concerns, organizations improve competitiveness, attract customers and investors, enhance employee morale and productivity, mitigate risks, and foster long-term profitability.

CSR practices encompass a wide range of activities and initiatives that organizations undertake to demonstrate their commitment to societal well-being. These practices include: (a) *Environmental Sustainability:* Organizations can adopt environmentally friendly practices by reducing waste, promoting renewable energy, conserving resources, and recycling undertakings; (b) *Ethical Supply Chain Management:* Organizations can promote fair labor practices, ensure supply chain transparency, prevent human rights abuses, and support responsible sourcing and production processes; (c) *Philanthropy and Community Engagement*: Organizations can contribute to the community through donations, sponsorships, volunteering, and supporting local initiatives that address social challenges; (d) *Employee Well-being*: Organizations can focus on the well-being and development of their employees by providing a safe work environment, offering fair wages, providing training and development opportunities, work-life balance initiatives, and diversity and inclusion programs; and (e) *Ethical Governance:* Organizations can establish ethical codes of conduct, transparent governance practices, and mechanisms for addressing ethical concerns and whistleblowing.

Finally, we integrate the findings outlined above by presenting empirical studies that highlight the interplay between ethics, justice, CSR, and performance management and the positive outcomes thereof.

## Empirical findings that highlight the interplay between ethics, justice, CSR, and performance management

A review of the interplay between Ethics, Justice, CSR, Performance, and Performance Management, for the most part, reveals many positive outcomes, as indicated by a selection of studies illustrated below:

A meta-analysis by Orlitzky et al. (2003) found a positive relationship between CSR and financial performance across multiple studies, possibly a precursor of Turker’s (2009) indication that CSR practices positively impact employee attitudes and behaviors, including job satisfaction, organizational commitment and, ultimately, performance. Colquitt et al. (2001) found that ethical climate, distributive justice, and CSR climate significantly predict employee reactions to performance management processes, including satisfaction with the system, commitment to the organization, and job performance, a finding basically replicated by Aguinis and Glavas (2012), who highlighted the role of ethical climate and CSR in enhancing the effectiveness of performance management systems and employee performance outcomes. Ryan and Saha (2016) gave specific attention to diversity and inclusion in the promotion process and indicated that those procedures enhanced perceptions of organizational justice and employee satisfaction, confirming a previous meta-analysis by Joshi et al. (2011) indicating a positive relationship between diversity management practices and organizational justice perceptions. Tourigay et al. (2019) revealed that (1) ethical leadership relates positively to CSR at the work unit level, and (2) CSR has a positive cross-level effect on organizational trust at the individual level. The latter outcome positively impacted on organizational citizenship behavior through the mediating effect of taking personal social responsibility.

## Caveats

### More on ethical leadership and CSR

In this context and following a systematic review of 114 academic papers about ethical leadership, CSR, and their impact on firm PM over 50 years until 2016, Saha et al. (2019) indicated that research in recent years has increasingly focused on ethical leadership.

Saha et al. (2019) claimed that ethical leadership is a fundamental requisite for organizations wishing to set apart their companies from competitors in terms of both CSR and performance aspects (Yoona and Chung, 2018; Luque and Herrero-García, 2019). Furthermore, Saha et al. (2019) stressed the “personal values impact” on ethical leadership that directly impacts CSR initiatives and directly and indirectly impacts firm performance toward achieving a competitive edge (e.g., De Roeck and Farooq, 2018; Kim and Thapa, 2018).

Given our previous comments on the distinction between the financial end-product and utilitarianism, it is germane to note that Saha et al. (2019) distinguished between CSR programs focused on achieving an enhanced financial bottom line and those orientated ostensibly on socially oriented changes and initiatives. Notably, we have established that leaders high on EL serve as role models and promote socially responsible behavior among employees (Brown et al., 2005; De Hoogh and Hartog, 2008). However, it appears, furthermore, that ethical leadership and initiatives in the workplace impinge not only on workers’ productivity but also, following Saha et al. (2019, p. 435), an ethical leadership style (EL) fosters social initiatives and that precisely, those highly engaged leaders with high-level EL are the drivers of CSR programs. Moreover, within the context of our comments on institutionalized ethics, it is refreshing that researchers have acknowledged that such high-level EL leaders “care more about their employees, their firm, and society than their self-interest (Brown and Treviño, 2006).”

Ultimately, and perhaps precisely because of the utilitarian or philosophical approach, following this line of discussion, strong ethical leadership creates a win-win situation for all the stakeholders involved. Indeed, other firm advantages accrued by high ethical leadership in CSR programs include high-level interactions between management and employees, strong relationships with shareholders that reduce sustainable risk factors, and improved firm competencies (Rettab et al., 2008). In sum, Saha et al. (2019) maintain that CSR is positively related to the three components of organizational performance: financial, employee commitment, and corporate reputation (p. 436). Indeed, following Wang et al. (2015), ethical and sustainable leadership earns companies more external legitimacy and positive brand images because of their (socially) responsible business practices.

While the picture painted of CSR and the advantages of a fair and well-directed ethical stance toward shareholders and society have been documented, Saha et al. (2019) astutely indicate that internal and external market forces mitigate against firms not “mature” enough to formulate and implement a strategic plan and integrated organizational culture to implement CSR strategies (Dobers and Halme, 2009). Moreover, after Vashchenko (2017), we concur that firms are less likely to engage in socially responsible practices if the inflation rate is high, consumer confidence is weak, and productivity growth is low.

Finally, Saha et al. (2019, p. 436) assert that their findings and propositions “bridge the gap between past and future research.” In their parlance, we are left with the following thoughts to ponder, namely, (a) in the organization, how personal values affect EL; (b) how the internal and external environment influences the adoption of CSR practices; (c) the extent to which CSR project costs negatively affect the adoption of CSR practices; (d) the positive impact of EL on CSR; (e) EL’s direct and indirect positive impact on firm performance (FP); and (f) the extent to which CSR has a direct positive impact on FP.

The findings being as they are, the researchers conclude that willy-nilly in today’s global competition, firms must meet their stakeholders’ and society’s expectations and interests if they are to survive. Moreover, they assert categorically that firms need ethical leadership “rather than pure leadership” to achieve their sustainable strategies (p. 436). Saha et al. (2019) concluded by reminding managers everywhere that, with considerations of organizational justice at the forefront of organizational policy and strategy, moral standards, role clarification, knowledge, and commitment constitute the backbone of ethical and responsible leadership that will stimulate today’s employees to collectively support the CSR practices that retain employees, customers, and other stakeholders.

### CSR and the CSR gap: the distinction between internal and external CSR

We have had reason to relate so far to ethical leadership as it relates to outcomes (a) within the organization and (b) external to the inner workings of the firm, insofar as their effects impinge upon a variety of stakeholders and society in general. Returning to the relationship between CSR, firm performance, and organizational justice, Cao et al. (2023) go beyond linear relationships between perceived justice and work outcomes to posit that the gap between CSR activities that benefit internal stakeholders (e.g., employees; Scheidler et al., 2019) and those on behalf of external stakeholders (e.g., business partners and the local community) clash at the expense of the employees with consequent negative outcomes for the latter group (and ultimately to the firm) because of the company’s resource limitations (Rupp et al., 2006).

As observed above, organizational justice is linked closely to employee performance—and CSR-related activities are no exception. For instance, Cao et al. (2023, p. 1) noted the utility of OJ as a tool to measure relationships between CSR and employees’ behaviors (e.g., Rupp et al., 2006; De Roeck et al., 2014a; Chen and Khuangga, 2021). A distinction is made between *internal* CSR, related to employees’ *direct* interests (e.g., training, care of employees, Farooq et al., 2017) and *first-party justice* (Chen and Khuangga, 2021), while *external* CSR caters to CSR activities, such as charitable donations, environmental protection investment, and customer care (*third-party justice*), more concerned with society, the environment, and business partners (Deng et al., 2019).

In their investigation, Cao et al. (2023) posited that the CSR gap leads to employees’ perceptions of *injustice*, thus reducing the firm’s efforts and damaging firm performance. Specifically, the authors argue that CSR disclosure of information about an organization’s activities (investments) and the interpretation of such data by employees as an injustice can amplify the negative impact of the CSR gap. Exposure to information such as the Employee Stock Ownership Plan, however, reduces that negative effect because it serves employees’ direct interests.

Cao et al. (2023) also noted that internal and external activities, respectively, generally work to the advantage of CSR-oriented firms by improving their image, providing access to sources, and even serving as a rationale for an organization’s “behavior.” Much depends on how one interprets such actions, and employees’ perceptions are no exception, especially when internal and external processes clash. However, even on an “objective” level, the authors indicate that primarily because of the extreme investments involved in CSR activity, the gains do not always cover the losses.

Furthermore, Cao et al. (2023, p. 2) indicate that (recent) inconsistent empirical evidence on the impact of CSR on organizational justice might be explained by the failure to consider the CSR gap. The authors cite De Roeck et al. (2014b), who indicated that employees’ high perception of organizational justice was a function of correspondingly high levels of both internal and external CSR, contradicting Kim et al.’s (2021) study that revealed insignificant relationships between the variables. Additionally, Cao et al. (2023, p. 2) further cite Chen and Khuangga’s (2021) observation that while neither category of CSR had an impact on employee behavior, the *combination* of high levels of internal and external CSR did engender positive employee conduct.

Cao et al. (2023) argue that information available to employees from the Internet and media outlets concerning the CSR gap—increases employees’ subjective perception of injustice because they feel discriminated against in favor of external stakeholders’ CSR investments. Consequently, Cao et al. (2023) assert that in line with the research on injustice, the outcome reveals itself in reduced organizational commitment and higher turnover (e.g., Kim et al., 2021) with all the concomitant effects on performance.

Notably, the findings of this study raise an interesting conundrum indicating that external CSR investments are a “double-edged” sword. While the CSR gap is relatively small, internal stakeholders can live with the company’s external investments and even take pride in them, but when the external CSR investment produces a wide CSR gap, it likely triggers their negative responses. In the authors’ words, the results seemingly “deny” the positive role of external CSR because firms would benefit most when there is high internal but no external CSR (p. 8). However, this study concludes that at a certain total CSR investment (controlling for the effects of total CSR investments), investing in internal CSR is more effective than investing in external CSR for improving firm performance.

The result is not surprising, however, when we consider that, ultimately, internal CSR perceptions of fairness and justice have a greater weighting in the eyes of employees than “operation-related” CSR, such as diversity, human rights, and community relations (Lee et al., 2013). From a practical point of view, management should be wary of overly flouting their “investments;” rather, they should find the appropriate balance between internal and external investments to maintain a contented and committed workforce that contributes to the firm’s financial success and contribution to society. Perhaps the lesson to be learned is that performance management is as much about managing management as it is about evaluating employees’ productivity levels.

## Practical measures for improving PM

Drawing on the theoretical underpinnings and empirical evidence, this section provides practical recommendations for organizations to promote ethics, justice, CSR, and effective performance management.

In an informative article, in the heat of the debate on the efficacy of PE systems, Grubb (2007) recommended that organizations galvanize staff at all levels to take a fresh look at their workplace and adapt innovative steps to enhance worker proficiency and morale, a position that the authors of this article endorse.

True, raters might have been trained to be (more) objective and unbiased, to provide clear performance expectations, evaluation criteria, and feedback mechanisms to employees, to provide clear performance expectations, evaluation criteria, and feedback mechanisms to employees, and to ensure that their PE processes are perceived as fair, transparent, and consistent (e.g., Bonner et al., 2000). However, in Grubb’s view, there needs to be a continuous process where employees cooperate with management and suggest measures for improvement; the overall participation of staff enhances the group spirit and loyalty to the firm (see also Iles and Judge, 2005). In such an environment, all shades of ethnic associations and worldviews are acknowledged and respected and attention is given to the needs and sensitivities of employees, especially in the multi-cultural work environment of the 21st century. Consequently, the firm gains a reputation as a caring employer that attracts and retains competent employees.

Incorporated into the many suggestions and echoing Tziner et al. (2015), Grubb proposed that a convenient place to start the process of reconstruction of the company’s ethos was the crafting of individual work contracts that itemize what “each side of the contract will do for the other.” Frequent feedback reviews replace performance evaluations (after Lee, 2016). Moreover, citing Buckingham (2005), the sessions focus on employees’ strengths and not on weaknesses and failures, on praise rather than admonishment. For their part, employees are urged to focus on organizational accomplishment rather than on other’s perceptions of their competence.

Supervisors thus become mentors who work on how their charges can overcome barriers and how they can set learning goals and strategies within the context of mutually agreed objectives rather than concentrating on performance outcomes. To that end, superiors are trained in communication skills that include encouraging all levels of staff to take responsibility for their progress and development at work. Consequently, Grubb asserts, workers’ self-efficacy, confidence, and motivation are enhanced (Margolis and McCabe, 2006).

Moving toward establishing an ethical culture in the workplace, honesty and fairness are key elements, as are openness and accountability that, as we have seen, impinge on employees’ perceptions of justice and their positive work attitudes and behavior. For instance, Grubb (2007) suggested that organizations strive toward advancement based on knowledge and skills that fit tasks instead of the possible subjective perceptions of a senior member of staff. In that respect, Grubb advocates for multi-observers from different backgrounds and perspectives to assess candidates for advancement. Moreover, Grubb proposes that organizations move toward an operation that is flatter, where advancement is less dependent on promotion along a hierarchy. This proposition aligns itself with the emerging pattern of job mobility as described by Rabenu (2021) and further lessens the likelihood of political interests intruding on promotion decisions.

Focusing on the establishment of an ethical culture, we would encourage firms to foster an organizational culture that consists of a set of shared values and norms that emphasize ethical behavior (Victor and Cullen, 1988a,b). This can be achieved through leadership commitment, communication of ethical values, and providing ethics training and resources to both leaders and employees (Mayer et al., 2009). Ethical decision-making is achieved by the establishment of clear guidelines and integrating ethical considerations into performance evaluation criteria. Moreover, the current digital era facilitates providing objective data-driven insights into employee performance leading to the reduction of unbiased, fair, and equitable rewards for employees. The performance feedback is continuously accessible, thereby allowing for transparent knowledge of strengths, weaknesses, and performance facets requiring improvement (Curzi et al., 2019).

Turning to the integration of CSR into performance management, we recommend aligning performance goals with CSR objectives to highlight the role that the firm attaches to such issues as social responsibility and sustainability. Notably, Rupp et al. (2006) noted the significant impact of corporate social responsibility on employee commitment and performance. Employees and groups of employees can and should be recognized for demonstrating social responsibility initiatives, contributions toward sustainable practices, and for demonstrating ethical behavior. In that respect and given the level of importance we have attached to ethical leadership, management is urged to enhance training for all levels of senior staff regarding ethical leadership practices and their role in promoting ethical conduct and fairness in all aspects of the organization’s dealings, including performance management.

We close by reiterating that along with Victor and Cullen (1988a,b), we stress the moral implications that ethical leadership bestows on an organization and its stakeholders insofar as the company is honest, fair, and just in all its dealings with employees and investors alike. We nevertheless recognize, along with the research, that ethical leadership is associated with several significant outcomes beyond the narrower concerns of employee satisfaction, such as innovation, profitability, and customer satisfaction and commitment (Brown et al., 2005).

By implementing these practical measures, organizations can enhance their sustainable performance management processes, promote ethical behavior, fairness, and social responsibility, and create a positive work environment that fosters employee engagement, growth, and organizational success.

## Author contributions

AT: Writing – original draft. MP: Writing – review & editing.
